# Effect of Cactus (*Opuntia ficus-indica*) and Acacia (*Acacia seyal*) Gums on the Pasting, Thermal, Textural, and Rheological Properties of Corn, Sweet Potato, and Turkish Bean Starches

**DOI:** 10.3390/molecules27030701

**Published:** 2022-01-21

**Authors:** Shahzad Hussain, Abdellatif A. Mohamed, Mohamed S. Alamri, Mohamed A. Ibraheem, Akram A. Abdo Qasem, Tawfiq Alsulami, Ibrahim A. Ababtain

**Affiliations:** Department of Food Science and Nutrition, College of Food and Agricultural Sciences, King Saud University, Riyadh 1145, Saudi Arabia; abdmohamed@ksu.edu.sa (A.A.M.); msalamri@ksu.edu.sa (M.S.A.); mfadol@ksu.edu.sa (M.A.I.); aqasem@ksu.edu.sa (A.A.A.Q.); talsulami@ksu.edu.sa (T.A.); ababtain.ibr@gmail.com (I.A.A.)

**Keywords:** cactus, acacia, gums, corn, sweet potato, Turkish beans, starch, pasting, thermal

## Abstract

This study was planned to explore the locally available natural sources of gum hydrocolloids as a natural modifier of different starch properties. Corn (CS), sweet potato (SPS), and Turkish bean (TBS) starches were mixed with locally extracted native or acetylated cactus (CG) and acacia (AG) gums at 2 and 5% replacement levels. The binary mixtures (starch–gums) were prepared in water, freeze dried, ground to powder, and stored airtight. A rapid viscoanalyzer (RVA), differential scanning calorimeter (DSC), texture analyzer, and dynamic rheometer were used to explore their pasting, thermal, textural, and rheological properties. The presence of acetylated AG or CG increased the final viscosity (FV) in all three starches when compared to starch pastes containing native gums. Plain SPS dispersion had a higher pasting temperature (PT) than CS and TBS. The addition of AG or CG increased the PT of CS, SPS, and TBS. The thermograms revealed the overall enthalpy change of the starch and gum blends: TBS > SPS > CS. The peak temperature (*T_p_*) of starches increased with increasing gum concentration from 2 to 5% for both AG and CG native and modified gums. When compared to the control gels, the addition of 2% CG, either native or modified, reduced the syneresis of starch gels. However, further addition (5% CG) increased the gels’ syneresis. Furthermore, the syneresis for the first cycle on the fourth day was higher than the second cycle on the eighth day for all starches. The addition of native and acetylated CG reduced the hardness of starch gels at all concentrations tested. All of the starch dispersions had higher G′ than G″ values, indicating that they were more elastic and less viscous with or without the gums. The apparent viscosity of all starch gels decreased as shear was increased, with profiles indicating time-dependent thixotropic behavior. All of the starch gels, with or without gums, showed a non-Newtonian shear thinning trend in the shear stress vs. shear rate graphs. The addition of acetylated CG gum to CS resulted in a higher activation energy (*Ea*) than the native counterparts and the control. More specifically, starch gels with a higher gum concentration (5%) provided greater *Ea* than their native counterparts.

## 1. Introduction

Long-chain polymers known as hydrocolloids (gums) are widely used in the food industry. Fluid biopolymers with high molecular weight, such as food hydrocolloids, can be used as functional ingredients for improving product texture, shelf life, and microstructure [1]. Polysaccharides that make up the majority of gums are long-chain hydrophilic molecules with high molecular weights [2]. In addition to thickening, gelling, water retention, dispersing stabilizers, foaming agents, and texture modifiers, gums can also be used as flavoring agents. Hydrocolloid gums are most commonly used in processed foods such as bread, cakes, mayonnaise, dressings, jellies, dessert, ice cream, gravies, and soups [3].

Food hydrocolloids are increasingly being used in the baking industry to achieve a variety of functionalities. To achieve the desired synergy between specific functional properties, a variety of gums can be used. The flour mixtures can be made texturally stable, freeze-thaw stable, and age resistant by adding gums before baking [4,5]. Gums can improve bread’s water-holding capacity, texture, volume, uniformity of cell structure, and product quality during storage, among other qualities [6].

When used as a gluten substitute in gluten-free bread formulations, hydrocolloids can also alter the properties of starch [7,8,9]. Baked goods can also benefit from the elastic properties of gums, which can be softer with low gums concentration or harder with higher concentration when stored at room temperature or in the refrigerator [10]. Food products that contain water-soluble gums are considered to be good sources of soluble dietary fibers. Gums can help control cholesterol and glucose levels in the blood when used in functional foods or as nutraceuticals of various types [11]. 

The gums are exploited as an alternate way for chemical and enzymatic starch modification; a generally safe and low-cost technique of starch modification. Gums are derived from a variety of sources. Plant exudates (gum arabic, gum karaya, gum ghatti, gum tragacanth), seeds (guar gum, locust bean gum, tara gum, tamarind gum), tubers (konjac mannan), algal (agar, carrageenan, alginate), and microbial (xanthan gum, curdlan, gellan gum) are the most frequent [12]. There are several more plant gum sources, including flaxseed, arabic gum from acacia plant, okra gum, and cordia. In the food processing industry, hydrocolloids such as alginates, carrageenan, agar, guar gum, gum arabic, and carboxymethyl cellulose are well-known stabilizers [13]. The thermal and pasting capabilities of many types of starches are greatly influenced by gum hydrocolloids such as okra, guar, xanthan, flaxseed, gellan, and carrageenan [14,15]. Pasting, thermal, textural, and rheological properties of rice, sorghum, wheat, chickpea, potato, sweet potato, and corn starches were significantly affected in the presence of 5, 10, and 15% okra gum [16,17].

Acacia gum, also known as gum arabic, is an exudate from the acacia tree’s stems and branches. It is ground, dissolved in water, filtered, heated, and then spray or drum-dried [12]. Galactose (39–42%), arabinose (24–27%), glucuronic acid (15–16%), and rhamnose (12–16%) make up the bulk of its chemical composition [18]. Unlike other gums, arabic gum can yield a clear aqueous solution as high as 50% (W/W). Viscosity decreases when the pH of the solution drops below 4.5 (the ideal pH for this solution is between 4.0 and 5.0), whereas electrolytes results in drop of its viscosity [12].

Natural gums are preferred because they are less expensive, more readily available, and less hazardous than the currently used methods (e.g., chemical, enzymatic) of starch modification [19]. Hydrocolloids can be obtained from locally grown cactus and acacia plants, according to the proposed research. Arabic gum (acacia) is widely studied, but cactus (cactus gum) has not received the same attention. Cactus gum is a water purification agent that works by lowering turbidity, reducing suspended particles, and removing heavy metals [20,21]. The present work was accomplished to explore the effect of gums extracted from local sources on the properties of different starches, such as their pasting, thermal, and rheological behaviors. 

## 2. Materials and Methods

### 2.1. Collection of Raw Material

Fresh cactus cladodes and acacia gum exudates were collected from the Dirab Agriculture Research Station of King Saud University (Riyadh, Saudi Arabia). The raw plant materials were then transported to the lab for further processing and use in experimental work. Commercial corn starch was purchased from ARASCO (Riyadh, Saudi Arabia). 

### 2.2. Isolation of Turkish Bean and Sweet Potato Starches 

Turkish bean (TB) meal and water were mixed together in a heavy-duty blender for about five minutes. The slurry was poured through a 200-mesh sieve. The filtrate was then centrifuged for 15 min at a speed of 2000× *g* to separate it. Afterward, the layer on top was removed, and the white pellet at the bottom was re-suspended in distilled water and centrifuged at the same speed as before. This process was repeated. Finally, a white pure starch fraction was made after this process was done five times. The sweet potato tubers were carefully washed and skinned, then slurry was made by blending the diced tubers and distilled water (50/50) for 3 min in a heavy-duty blender. A muslin cloth was used to filter the resulting sludge. After that, the residue was re-suspended in distilled water (1:2 *v*/*v*) and filtered again. The filtrates were filtered once more via a 200-mesh sieve. The starch was allowed to settle at room temperature for 3 h before the supernatant was removed. The starch was re-suspended in distilled water and centrifuged for 15 min at 2000× *g*. The darkish waxy layer on top of the bottle was removed after centrifugation, and the white material at the bottom of the bottle was reconstituted with distilled water and centrifuged until pure white starch portion was obtained. The starch was then dried in acetone and pulverized in a coffee grinder. The starch powder was then placed in airtight glass bottles and kept in the freezer until needed.

### 2.3. Isolation of Cactus Gum

Cactus cladodes were washed and diced properly before being blanched in 80 percent ethanol to inactivate any enzymes present. They were then blended in 1:3 ratios with distilled water for 1 min before being filtered through muslin cloth and centrifuged at 5000× *g* for 15 min to extract the cladodes gum. After centrifuging the material again, the supernatant was collected, neutralized, and freeze dried. Before being stored in airtight jars under refrigeration, freeze dried gum was pulverized into powders, passed through a 60-mesh screen, and labeled with the code CG [22].

### 2.4. Preparation of Acacia Gum Powder

Acacia gum nodes that had been taken from the plants were cleaned and dried in the shade. The dried nodules were ground with a heavy-duty grinder to make them into powder. They were then sieved through 60 mesh, coded as AG, and stored in airtight jars until they were used again.

### 2.5. Gums Modification

Freeze dried CG or AG were mixed with water at 10% (*w*/*v*) for 60 min at 40 °C. A total of 0.50 N sodium hydroxide was used to keep the pH between 8 and 8.5. Drops of acetic anhydride were added to the water, while the pH was kept between 8 and 8.5. At the end of the reaction, the pH of the contents was changed to between 6.0 and 6.5. The final product was freeze dried, passed through a 60-mesh sieve, and stored in airtight jars.

### 2.6. Fourier Transform Infrared (FTIR) Analysis of Native and Acetylated Gums

The Alpha-ATR-FTIR spectrophotometer (Bruker Alpha-Eco ATR-FTIR; Bruker Optics Inc., Ettlingen, Germany) was used to get the spectra of both natural and modified gums. This was done with a scan speed of 1 cm/s and a resolution of 4 cm^−1^. In the absorption mode, the samples were analyzed in the frequency range of 400 to 4000 cm^−1^

### 2.7. Preparation of Starch Gum Mixtures

At 2 and 5% replacement levels, cactus gum (CG) and acacia gum (AG) powders (native or acetylated) were combined (in triplicate) with corn, sweet potato, and Turkish bean starches. To obtain homogeneous distribution of gum in the starch, the wet blending method was applied. The starch-gum mixtures were combined with water (1:2), thoroughly stirred, and then freeze dried. The freeze-dried mixtures were ground to a fine powder and stored in airtight jars until required.

### 2.8. Pasting Properties of Starch Gum Mixtures

Rapid Visco analyzer (RVA, Newport Scientific, Sydney, Australia) was used to study the pasting properties of starch-gum mixtures. Native starches or starch-gum mixtures (3 g at 14 percent moisture basis) were poured into aluminum canisters and distilled water was added to attain a total weight of 28 g. The slurry was heated to 95 °C in 4.40 min (at 10.23 °C/min), incubated for 4 min at 95 °C, and then cooled to 50 °C in 4.40 min (at 10.23 °C/min) and incubated for 2 min at 50 °C. Thermocline window software, which came with the device, was used to process the data for pasting parameters like peak viscosity, final viscosity, setback viscosity, and pasting temperature.

### 2.9. Differential Scanning Calorimetry (DSC)

The thermal behaviors of starch-gum mixtures were investigated using DSC (Q 2000, TA instruments, New Castle, DE, USA). In aluminum pans, samples (6–8 mg) and 10 µL distilled water were added. The pans were hermetically sealed and left to equilibrate for 4 h at room temperature. As a reference cell, an empty pan was employed. Thermal scanning of sealed samples was carried out at temperatures ranging from 25 to 110 °C/min at a rate of 10 °C/min. The Universal Analysis Software was used to determine thermal transitions such as enthalpy ∆H (J/g), onset temperature (*T_o_*), and peak temperature (*T_p_*).

### 2.10. Freeze Thaw Stability of Starch Gels

The rapid visco analyzer (RVA) was used to cook starch gels (3 g starch, 14 percent moisture basis, dissolved in water to reach total 28 g weight) as described in Section 2.8. Cooked starch gels were placed in tubes and kept in the freezer at −20 °C. After 4 days, frozen gels were removed and thawed in a hot water bath at 50 °C for 20 min. The water isolated from the gels was measured after centrifugation at 3000× *g* for 15 min. The gels were then frozen for four more days before going through another freeze-thaw cycle. Percent syneresis was used to describe the separation of water from gels after 4 and 8 days of storage.

### 2.11. Textural Profile Analysis of Starch Gels

Starch gels were retained in aluminum containers (35 mm height, 30 mm internal diameter) and stored at room temperature overnight after the RVA experiment (3 g starch at 14 percent moisture) as indicated in Section 2.8. The gels were compressed in two penetration cycles at a speed of 0.5 mm/s to a distance of 10 mm using a Brookfield CT3 Texture Analyzer (Brookfield Engineering Laboratories, Inc. Middleboro, MA, USA) fitted with a 12.7 mm broad and 35 long cylindrical probe. Textural parameters including gel hardness, springiness, cohesiveness, and adhesiveness were directly measured, while chewiness was computed as a product of gumminess and springiness.

### 2.12. Dynamic Rheological Properties of Gels

The dynamic rheological properties of cooked gels were studied by using a TA Discovery Hybrid Rheometer (New Castle, PA, USA) equipped with a cone-plate measuring system (2° angle, 40 mm in diameter). Following the calculation of the linear viscoelastic area, a constant strain of 5% was applied. Using frequency sweeps ranging from 0.1 to 100 rad/s, the storage moduli (G′), loss moduli (G″), and dynamic mechanical loss tangent (tan = G″/G′) were determined at 30 °C.

### 2.13. Steady Shear Behaviors and Temperature Dependency of Starch Gels

The flow curves were determined at 30, 40, and 50 °C with shear rates ranging from 0 to 100 (1/s) using a TA Discovery Hybrid Rheometer (New Castle, PA, USA) equipped with a cone-plate measuring system (2° angle, 40 mm in diameter). The starch-gum mixtures (5% *w*/*v*) were cooked in RVA under the same experimental conditions as described in Section 2.8. Cooked gels were transferred to a plate and geometry gap was adjusted to 100 µm, the excess sample was trimmed off with a spatula. The power law model (Equation (1)) was used to describe the experimental curves.

(1) $$Τ=K_{\gamma }^{n}$$

where τ = shear stress (Pa), *K* = consistency coefficient (Pa·s^n^), γ = shear rate (s^−1^), and *n* = flow behavior index (dimensionless).

The Arrhenius equation was used to calculate temperature dependence (Equation (2)).

(ln*μa* = ln*μ^o^* + *Ea*/RT) (2)

where *µa* = apparent viscosity, *µ_o_* = frequency factor, R = gas constant, *Ea* = energy of activation, T= absolute temperature (K)

The frequency factor is a constant that varies from reaction to reaction and relates the frequency of colliding molecules with enough energy to start a reaction. Temperature reciprocals (30, 40, and 50 °C converted to Kelvin as 303.15, 333.15, and 323.15, respectively) were plotted against the natural log of *K* values. Following the linear regression, the slope was multiplied by the universal gas constant (R = 8.314 J/mol·K) to obtain the value of *Ea*, and the inverse log of the intercept was used as *µ_o_* = frequency factor.

### 2.14. Statistical Analysis

All measurements were carried out in triplicate to ensure accuracy. A one-way analysis of variance (ANOVA) and Duncan’s Multiple Range (DMR) test at a *p* ≤ 0.05 level of significance was performed on the data using PASW^®^ Statistics 18 software to compare means.

## 3. Results and Discussion

### 3.1. FTIR of the Modified and Native Gums

**Figure 1:**
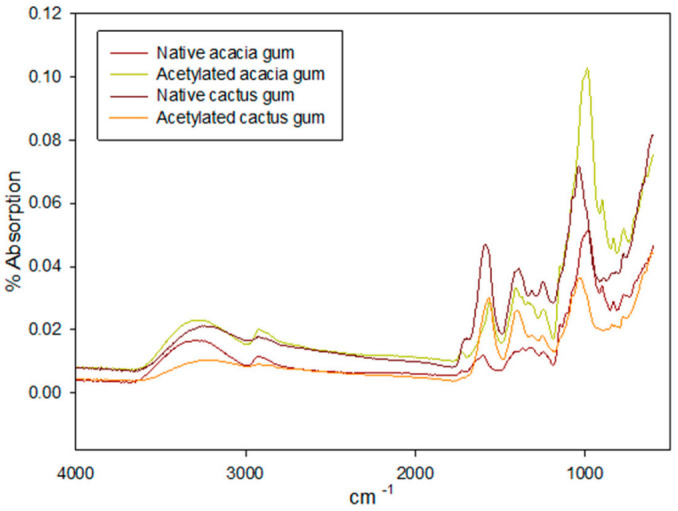
FTIR spectra of native and modified acacia and cactus gums.

The chemical structure of gums was evaluated for possible substitution of the acetyl functional group via FTIR analysis to verify the success of the acetylation of AG and CG, and the spectra are shown in Figure 1. A very strong but broader peak at 3395–3382 cm^−1^ was observed in native AG and CG, indicating -OH stretching vibrations, whereas the intensity of this peak was reduced in acetylated gums, indicating OH reduction after acetylation. Between 2927 and 2940 cm^−1^, a smaller but narrow peak was observed, indicating asymmetric stretching vibration of aliphatic -CH_3_ of the acetyl and methyl esters in polysaccharide gums [23]. The acetylated CG had the highest absorption for this peak, indicating that it was better substituted than AG (Figure 1). The C=O stretching vibrations in esterified carboxylic groups in gums are responsible for the minor shoulder peaks at 1721–1728 cm^−1^ [24]. The absence of any absorption peak in the acetylated AG or CG spectra around 1760–1800 cm^−1^ suggested that no unreacted acetic anhydride remained in the gums. The acetylated CG had a higher absorption band than the AG, indicating that the gums had been modified. Another intermediate-intensity peak was observed for the native and acetylated AG and CG, representing the characteristic deformations for NH in primary amines. Andrade, et al. [25] reported such findings in CG gums, claiming the presence of protein fractions in the gums. According to Osman et al. [26], the protein proportion in AG ranges from 0.03 to 2.8 percent. However, red shift was observed in acetylated AG and CG, where the peak shifted to lower wavenumbers. The FTIR spectral segment from 1500 cm^−1^ to downward wavenumber is referred to as the ‘fingerprinting region’. Because of the complex interacting vibration systems in this region, assigning an absorption peak to a precise vibration is difficult [27]. The small but sharp bands centered around 1415–1400 cm^−1^ are consistent with the symmetric stretching of COO of the carboxylate groups in the gums, whereas the peaks between 1310 and 1230 cm^−1^ are consistent with C-O stretching of the carboxylic acids [28]. Peaks between 1200–1000 cm^−1^ indicate C-O-C and secondary OH of the gums [29]. Thus, the FTIR spectra of both gums (AG and CG) show that they are primarily polysaccharides with minor fractions of associated proteins. The presence of C-O, C=O, and OH functional groups in AG and CG, on the other hand, determines their carbohydrate nature.

### 3.2. Pasting Properties of Starch-Gum Mixtures

**Figure 2:**
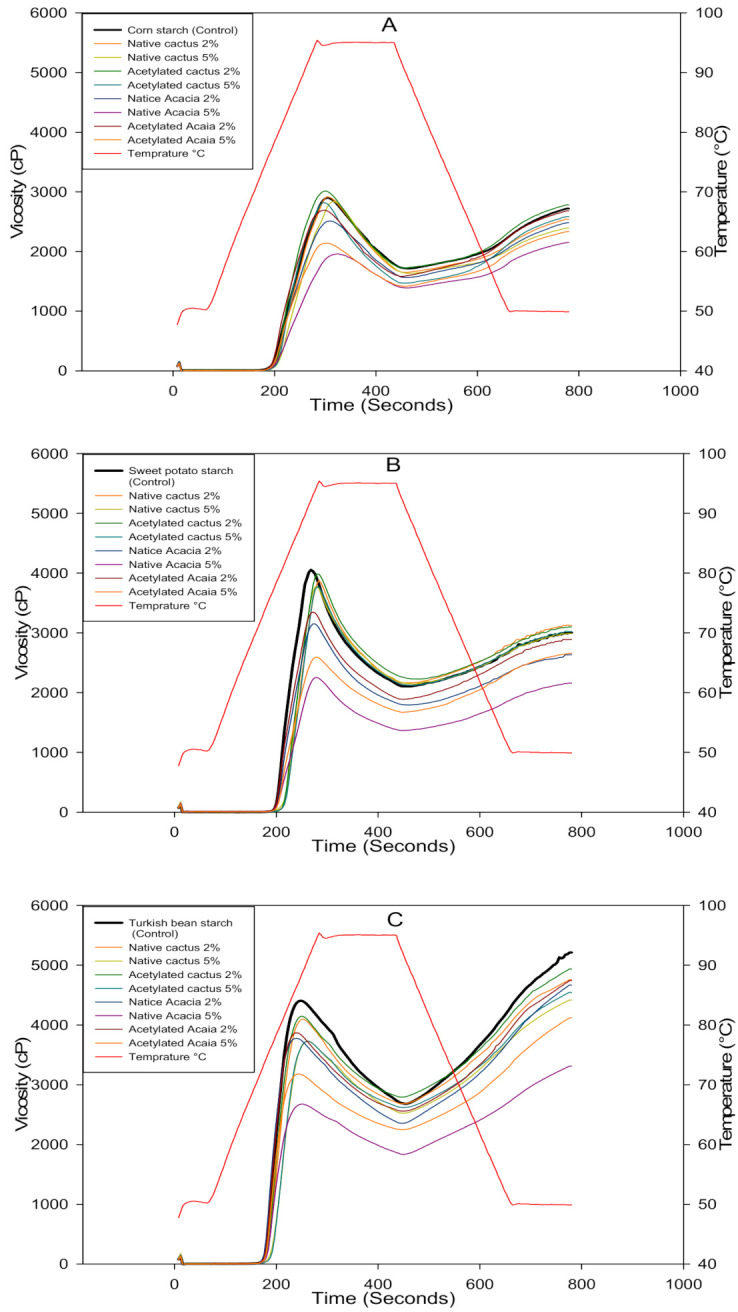
RVA profiles of corn (**A**), sweet potato (**B**), and Turkish bean (**C**) starches blended with cactus and acacia gums.

A rapid viscoanalyzer was used to estimate the pasting properties of CS, SPS, and TBS mixtures with native and acetylated gums, and the results are shown in Table 1 and Figure 2. The peak viscosity (PV) of starch indicates the maximum swelling caused by heat in the presence of water. TBS paste had the highest PV among the starches, followed by SPS and CS. This could be attributed to the TBS’s larger average granular size and swelling ability. However, when gums were added to the CS pastes, the highest PV was observed for CS with the addition of 2% acetylated CG, whereas a significant reduction in it was observed with increasing the gum to 5%. The addition of AG to CS, on the other hand, reduced the PV of the paste. However, a significant reduction in PV was observed at a higher percentage (5%) of AG in the CS paste. Similarly, to CS, the presence of AG or CG, either native or acetylated, reduced the PV of SPS and TBS compared to the control, with the effect being more pronounced when the gum concentration was increased to 5% in the paste. Overall, the addition of gums (5%), whether native or modified, had a negative effect on the PV of the starch pastes. Furthermore, when compared to their native counterparts, acetylated AG or CG provided significantly higher PV for all starches. The reduced PV could be attributed to the limited swelling of the starch granules in the presence of AG or CG gums, where the gums coated the starch granules and inhibited swelling under heat [30]. When xanthan gum was added to tapioca starch, similar results of reduced PV were reported [31]. The final viscosity (FV) of a starch-gum mixture is the viscosity at the end of a holding period at 50 °C. The system’s leached amylose fractions, which rearrange and align, have a significant impact on the final viscosity. According to the PV data, the TBS starch pastes had the highest FV of any starch. When compared to the control, the SPS paste containing both native and acetylated CG increased the FV of the mixture. This indicates that the gums improved the gelling ability of the TBS starch. In the CS and SPS pastes, the presence of CG, whether native or acetylated, resulted in a significant (*p* < 0.05) reduction in FV. When 5% gums were added versus 2% native and acetylated gums, the decrease in FV was more pronounced. The presence of acetylated AG or CG improved the FV in all three starches when compared to starch pastes containing native gums. This suggests that in the presence of modified gums, amylose undergoes greater retrogradation. Another study found that adding acetylated gum cordia to wheat flour pastes improved their FV more than the native gum [32]. According to Byars, et al. [33], adding guar gum to navy bean starch increased its FV.

The setback (SB) is the difference in the FV viscosity and the breakdown viscosity attained during cooling of the starch pastes, whereas, the breakdown viscosity is achieved after the PV by continuous shear of starch dispersions under heat. Setback begins with the short-term recrystallization of amylose chains as the temperature is reduced from 95 to 50 °C. Higher FV pastes exhibited higher SB viscosities for the starch pastes. As a result, the TBS paste without the addition of gum provided the highest SB. The addition of native or acetylated AG or CG, on the other hand, reduced the SB viscosity of the TBS. In contrast, the CS and SPS pastes had higher SB viscosity than the control with the addition of CG and AG. In the presence of guar gum, von Borries-Medrano et al. [34] observed enhanced short-term retrogradation and higher SB of starches. Nonetheless, starch pastes containing acetylated gums, either AG or CG, had significantly higher SB values than pastes containing their native counterparts. This suggests that the modified gums favored amylose-amylose interaction and allowed for greater short-term retrograde during cooling. In a previous study, adding native gum cordia to wheat flour gels resulted in lower SB viscosities than acetylated gum [32].

Pasting temperature (PT) is the temperature at which the viscosity of the starch dispersion begins to rise due to swelling and rupturing of the starch granules during heating. SPS dispersion had a higher PT than CS and TBS. The addition of gums, AG or CG, to the starches, on the other hand, increased their PT. Among the starches, the SPS with the addition of 5% acetylated CG had the highest PT (79.96 °C), followed by the 5% AG. The control TBS without the gums, on the other hand, had the lowest PT (71.77 °C). When compared to the AG counterparts, starch dispersions with CG resulted in a greater change in PT. However, the starch dispersions containing acetylated gums showed a lower change in PT than the native gums, with a more significant change in PT observed at 5% gum addition. The increase in the PT of starches could be due to a lack of available water for starch swelling and gelatinization as a result of water absorption by the added gums. When compared to the control, the addition of AG or CG raised the PT of the starches by 2 to 3 °C. This implies that starch blends with gums would require a higher cooking temperature than native starches. In contrast to current findings, the addition of gum cordia to corn starch resulted in a decrease in the PT of the starch dispersions [35]

### 3.3. Thermal Properties of Starch-Gum Mixtures

The heat energy required to gelatinize the starch in the dispersion is referred to as enthalpy. The thermograms revealed the following overall enthalpy of the starch and gum blends: TBS > SPS > CS > (Table 2). The enthalpy of the dispersion varies with the size of the starch granules as well as the presence of moisture. As a result, the grain size of SPS is smaller than that of the other starches, resulting in a higher enthalpy. The addition of native CG, on the other hand, improved the enthalpy of the starches except the TBS; in particular, the native gums performed better than their acetylated counterparts. The presence of AG, whether native or modified, reduced the enthalpic heat of starch dispersions. Acetylated gums, on the other hand, caused significant changes in the enthalpy of the starch blends, indicating that the gum modification manipulated the interactions with starch. Gelatinization enthalpy was affected differently by the addition of acetylated and native gums. The acetylated gum addition resulted in an increase in enthalpy, whereas the native gums resulted in a decrease in enthalpy. The increase in enthalpy could be attributed to a decrease in water in the dispersion as a result of gum controlling the amount of water, which resulted in lower chain mobility for starch and increased the heat required to gelatinize starch. In terms of enthalpy reduction, the presence of gums resulted in a lower enthalpy of the dispersions due to starch dilution, particularly amylose [31]. Similarly, the addition of cactus mucilage to sorghum starch resulted in a significant increase in enthalpy (∆H) [36].

The temperature at which starch in dispersion begins to swell reversibly when heated is referred to as the onset temperature (*T*_0_) [31]. The *T*_0_ of all starch dispersions increased with the addition of AG or CG at both concentrations, 2 and 5%. (Table 2). However, among all the starches, SPS had the highest *T*_0_, while TBS with CG at the highest (5%) level of addition had the lowest. The difference in *T*_0_ was more pronounced for starch blends with CG versus AG, whether native or modified. The addition of CG improved the *T*_0_ for the CS, SPS, and TBS by 4.34, 3.67, and 1.93 °C, respectively. However, increasing the CG and AG (native or modified) from 2 to 5% resulted in a significant change in the *T*_0_ of the starches. Hussain et al. [35] also reported that increasing the level of gum cordia addition from 3 to 12 percent increased the *T*_0_ of starch.

**Figure 3:**
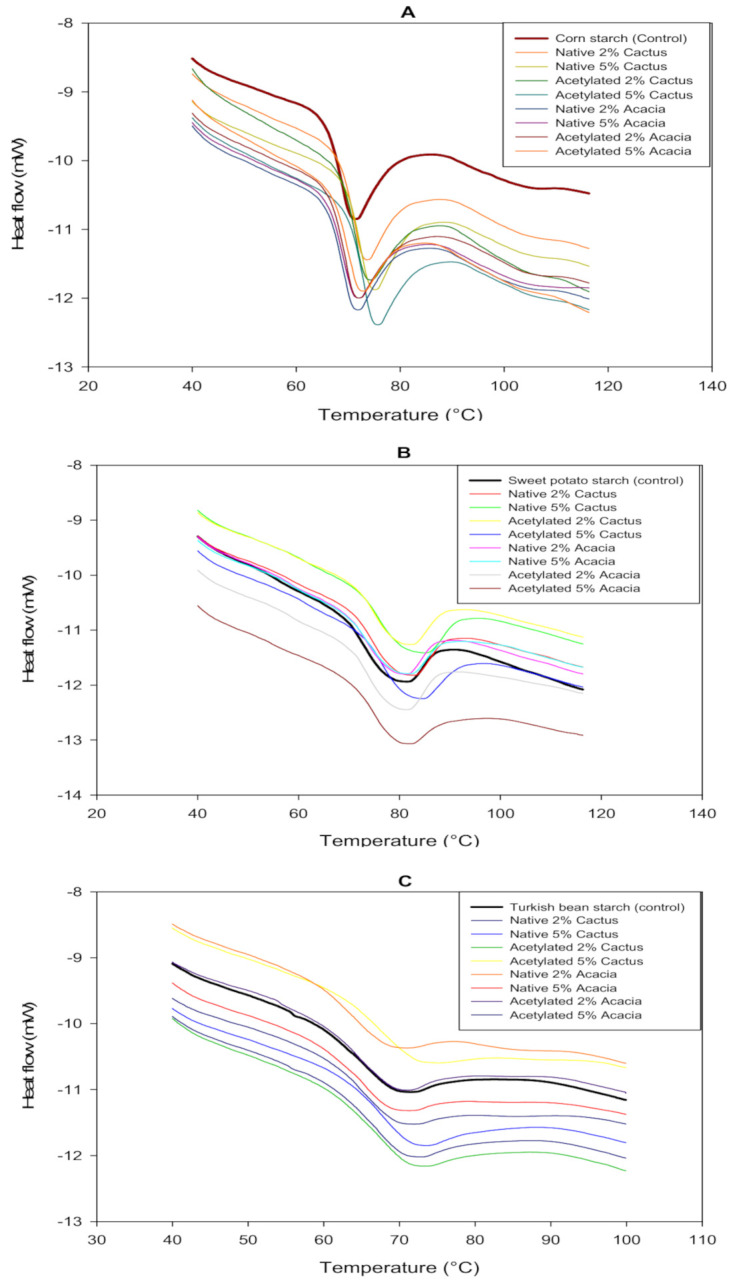
DSC thermograms of corn (**A**), sweet potato (**B**), and Turkish bean (**C**) starches blended with cactus and acacia gums.

Peak temperature (*T_p_*) is a moisture-dependent property of starchy foods that is estimated at the peak of starch gelatinization under applied heat. The *T_p_* of all starches increased significantly (*p* ≤ 0.05) when AG or CG were added. The *T_p_* of starch increased with increasing gum concentration from 2 to 5%, for both AG and CG native and modified gums. This increase in the *T_p_* supported the data of pasting temperature observed during the evaluation of pasting properties. The overall *T_p_* observed in the thermograms (Figure 3) was as follows: SPS > CS > TBS. The addition of CG, on the other hand, improved the *T_p_*, with a maximum rise of 4 °C observed for the CS blend containing 5% acetylated CG. This increase in *T_p_* could be attributed to the gum’s water binding ability, which reduced the amount of water available for starch granules, requiring a higher temperature to gelatinize starch [37]. In comparison to the AG, the CG improved the *T_p_* more than the AG. Furthermore, among the gums, acetylated CG or AG significantly increased the *T_p_* of starches. Rivera-Corona et al. [36] discovered a significant increase in the temperature of starch gelatinization with the addition of mucilage.

### 3.4. Freeze Thaw Stability of Starch-Gum Mixtures

Frozen foods containing starch are repeatedly freeze-thawed during storage and transportation. Thermal fluctuations in foods during transportation cause melting and re-crystallization of water, lowering the quality and acceptability. Simultaneously, the starch recrystallizes during cooling, resulting in water separation known as syneresis. Syneresis is an undesirable phenomenon that results in poor starch gel texture. In the current study, starch gels containing AG or CG were subjected to freeze-thaw cycles on the fourth and eighth days after initial freezing.

The highest syneresis was observed for CS gels, followed by SPS, and the lowest for TBS (Table 3). Under the given storage conditions, the syneresis for the first cycle at the fourth day was higher than the second cycle at the eighth day for all starches. The same thing happened when corn starch was frozen and thawed [35]. When compared to the control gels, the addition of 2% CG, either native or modified, reduced the syneresis of starch gels. However, further addition (5% CG) increased the gels’ syneresis. It implies that the starch/gum interaction was best at lower gum percentages rather than higher levels. It is hypothesized that at 2% CG, a favorable interaction between amylose and gum was established, which bound the water in the gel; however, at 5% CG, the thermodynamic interaction between gum molecules favored the formation of aggregated networks, which provided limited water binding. In all starch gels except TBS, acetylated CG provided lower wheying-off than native CG.

However, on the eighth day, all gels had lower overall syneresis than on the fourth day, with the exception of the TBS starch with CG (Table 3). Furthermore, with 5% CG, syneresis was observed to be greater than with 2% CG starch gels. This increase in syneresis during the second freeze-thaw cycle could be attributed to the deterioration of the cohesive gel matrix, particularly at higher gum concentrations where aggregates formed instead of a smooth gel structure [35]. However, the total syneresis of starch gels was lower at 2% CG and increased in the presence of 5%. Except for TBS gels, starch gels with native CG demonstrated higher syneresis than acetylated gels. This demonstrates that bean starch acted differently than cereal and tuber starches. It implies that TBS may be beneficial when no additives, such as gums and mucilages, are added to food gels.

Like CG, AG at 2% reduced the syneresis of all starch gels, with a significant increase in syneresis observed when the AG was increased from 2 to 5% on the fourth day. Except for the TBS gel, acetylated AG demonstrated greater syneresis than native AG in all gels. However, at a later ageing period of eight days, lower syneresis was observed for TBS gels, while no syneresis was observed for CS gels. In contrast to this study, two cycles of freeze-thawing for corn starch gels with cordia gum resulted in a higher syneresis [35]. In comparison to gels with acetylated counterparts, the presence of native AG in starch gels resulted in higher syneresis at all gum levels. In the case of total syneresis, a lower syneresis was observed at lower AG (2%) compared to control gels without AG, which increased as the gum level increased from 2 to 5%. Pongsawatmanit, et al. [38] found that increasing the xanthan gum mixtures and the number of freeze-thaw cycles resulted in a proportional increase in the syneresis percentage of tapioca starch.

When compared to CG, starch gels with AG resulted in greater total syneresis. In contrast to gels with higher gum concentrations, lower gum concentrations of either native or acetylated gum resulted in lower syneresis. Hussain et al. (2020) discovered that adding more gum cordia to corn starch gels increased syneresis. The gels containing acetylated gums, on the other hand, outperformed the native gums in terms of syneresis control. Several studies have shown that adding gums can improve the freeze-thaw stability of starch gels by lowering the syneresis [39,40].

### 3.5. Textural Properties of Gels Prepared from Starch-Gum Mixtures

The hardness of the starch gels is critical in determining the ease of mastication. The order of hardness among all the starch gels was as follows: SPS > TBS> CS > (Table 4). TBS’s higher hardness could be attributed to a faster rate of amylose recrystallization in the starch. Nonetheless, the addition of native and acetylated CG reduced the hardness of starch gels at all concentrations tested. Surprisingly, starch gels with a higher concentration of CG (5%) had lower hardness than gels with a lower concentration of CG (2%). For example, 5 percent CG in TBS gels resulted in a significant reduction in hardness (493 g) when compared to control TBS (852 g). However, starch gels containing native CG had lower hardness than gels containing acetylated CG. This increase in hardness could be attributed to phase separation in acetylated CG, which promoted greater interaction between leached amylose chains, resulting in recrystallization and higher gel hardness [41]. In a previous study, adding alkaline soluble okra gum increased the gel hardness of corn starch [17]. Except for the SPS, the addition of AG improved the control of the hardness of all starch gels and produced relatively softer gels when compared to CG. For example, the hardness of SPS gel was increased from 154 to 229 g with the addition of 5% native AG, whereas lower hardness (175 g) was observed with the same level of acetylated AG addition. The presence of acetyl functional groups in modified AG may have altered the level of interaction with leached amylose, resulting in reduced SPS gel hardness. The added gum may have hampered the interaction of leached amylose, resulting in lower interaction and, consequently, lower hardness [35]. This shows that the CG and AG behaved differently in controlling the starch gel hardness; however, native AG could produce softer gels. Higher hardness starch/gum blends are suitable for food products such as yogurt, ice cream, and confectionary [42,43].

The bond strength that makes up the body of the starch gel is indicated by cohesiveness. The cohesiveness of the starch gels remained between 0.33 and 0.71. (Table 4). The cohesiveness of the starch gels was greater in the case of SPS than in the cases of CS and TBS. Except for TBS, the addition of CG to the starch resulted in a decrease in the cohesiveness of SPS and CS. This variable effect could be attributed to the former two starches having higher amylose contents than the latter. Furthermore, the dilution of amylose in the presence of CG may be the cause of the gels’ decreased cohesiveness.

In a previous study, the addition of the gum Cordia increased the cohesiveness of wheat flour gels [32]. However, when compared to the gels with native counterparts, the acetylated CG had lower cohesiveness values for all of the starch gels. The reduced hydrogen bonding between starch and acetylated CG may have reduced the cohesiveness of the starch gels. In the case of AG, all starch gels except TBS gels showed a decrease in cohesiveness. In contrast to CG, acetylated AG interacted differently with the starch, resulting in higher cohesiveness than native AG gels. This difference in behavior between AG and CG could be attributed to inherent differences in the chemical structure of both gums, as AG contains minor protein fractions other than saccharides [44,45]. Previously, the inclusion of gum Cordia in corn starch gels improved the cohesiveness at all levels studied [35].

Gumminess is the product of the gels’ cohesiveness and hardness [17]. TBS had the most gumminess, followed by SPS, and then CS. The presence of CG reduced the gumminess of all of the starch gels. When compared to their native counterparts, acetylated CG was more effective at reducing gel gumminess. However, changing the concentration of CG from 2 to 5 percent in both the native and acetylated gums resulted in a significant (*p* ≤ 0.05) reduction in gumminess of starch gels. The reduction in gumminess was most noticeable in TBS, where the addition of 5% acetylated CG reduced gumminess from 284 to 230 g. The gumminess of AG was reduced when the concentration was increased from 2 to 5%, whether it was native or acetylated. The TBS gels containing 5% native AG showed the largest reduction in gumminess, with a decrease of 105 g. The structural alterations in the gums generated by acetyl substitution impacted the interactions between starch and AG, resulting in a considerable variation in the gumminess of starch gels with native and acetylated AG. Increasing the concentration of the gum cordia in wheat flour gel also reduces the gumminess of the gel, according to a previous study [32].

### 3.6. Dynamic Rheological Properties of Gels Prepared from Starch-Gum Mixtures

**Figure 4:**
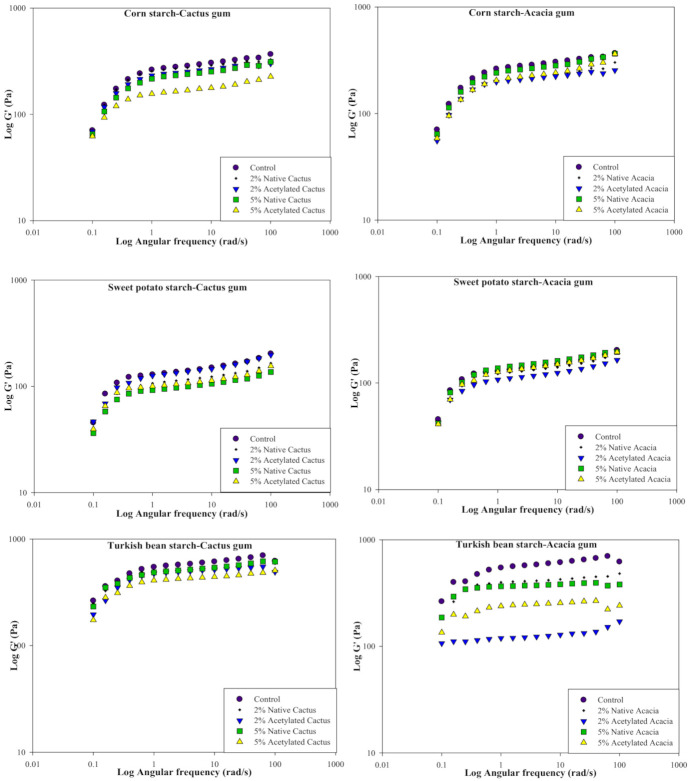
Effect of cactus and acacia gums on the storage modulus (G″) of corn, sweet potato, and Turkish bean starches.

Figure 4 depicts the storage (elastic) modulus (G′) of starch/gum dispersions. Before selecting the frequency sweep range of 0.1 to 100 (rad/s), the linear range of strain was estimated. All of the starch dispersions had higher G′ than G″ values, indicating that they were more elastic and less viscous. In the case of CS, the control had a higher modulus (G′) than all of the dispersions with native or modified cactus gum (CG) at all concentrations tested. For all dispersions, lower shear frequency caused a larger change in G′, especially at small deformation, whereas increasing frequency caused a smaller upshift in G′. The highest G′ was seen in the control CS dispersions, which decreased with the addition of 2% native or modified CG. However, at higher gum concentrations (5% acetylated), the dependence of G′ on frequency was observed to be minimal because the curve was close to the horizontal axis. Acacia gum (AG), like CG, provided a similar rise in G′ at small deformation for all dispersions, with the control remaining the highest among all samples. Flatter profiles were observed, as well as a lower dependence of G′ on frequency, particularly for dispersions containing higher concentrations of AG. Similarly, to CG, the acetylated AG showed the least change in G′ as the rate of deformation increased. However, when compared to the CG, the CS dispersion with AG showed a narrow physical gap between the profiles at the given rate of deformation change.

Similarly, without the addition of CG, SPS dispersions depicted better G′ than their counterparts. The addition of acetylated CG (2%) resulted in a similar dependence on the deformation rate. In dispersion, however, the least dependence of G′ on deformation was observed when 5% CG was added. However, acetylated CG outperformed the native CG in terms of G′. In contrast to the CG, the presence of a higher concentration of AG (5%) resulted in more elastic SPS dispersions, whereas the 2% AG showed the least dependence on deformation change. Thus, it shows that thermodynamic incompatibility did not favor intermolecular interactions between SPS and AG, but rather intramolecular interactions formed between molecules of the same type [46]. However, as the frequency was increased, the dispersions provided higher G′ for all of the gums, AG or CG.

TBS dispersion with CG resulted in a sharp rise in G′ at lower deformation rates, while relatively flatter plateaus were observed at higher frequencies. TBS, on the other hand, provided the highest G′ in the absence of CG. The addition of native CG reduced modulus dependence on frequency and left it less influenced, especially at higher deformation rates. This suggests that the addition of CG is not making a significant difference in the elastic properties of the starch dispersions. The superimposition of TBS dispersions with native CG (2 or 5%) indicated that both systems behaved similarly, especially at higher angular frequency. The acetylated CG, on the other hand, had the least effect on the TBS near the horizontal axis. It also implies that the system becomes independent of gum concentration and has a similar inner structure of the dispersion with comparable stability [47]. The TBS’s behavior with AG was found to be relatively inconsistent. The control without AG exhibited the greatest dependence on deformation rate and G′ compared to the other samples. However, the TBS with native AG produced similar profiles, whereas the acetylated gum was less influenced, with 2 percent remaining the least dependent and producing a straight line rather than downward curves. When compared to TBS with CG, the physical gap between all TBS/AG blends was much larger.

In general, the G′ trend for starches was TBS > SPS > CS. As a result, it implies that TBS could produce dispersions with improved elasticity and internal structure. However, because of the low dependency at higher frequencies, all of the starch dispersions with AC or CG could be considered weak gels. Such systems exhibit intermediate rheology between solution and gel, particularly at low deformation, while increasing deformation causes the three-dimensional network to break up into smaller clusters. Hussain et al. [35] observed an increase in G′ of the CS gels as the concentration of gum Cordia was increased.

**Figure 5:**
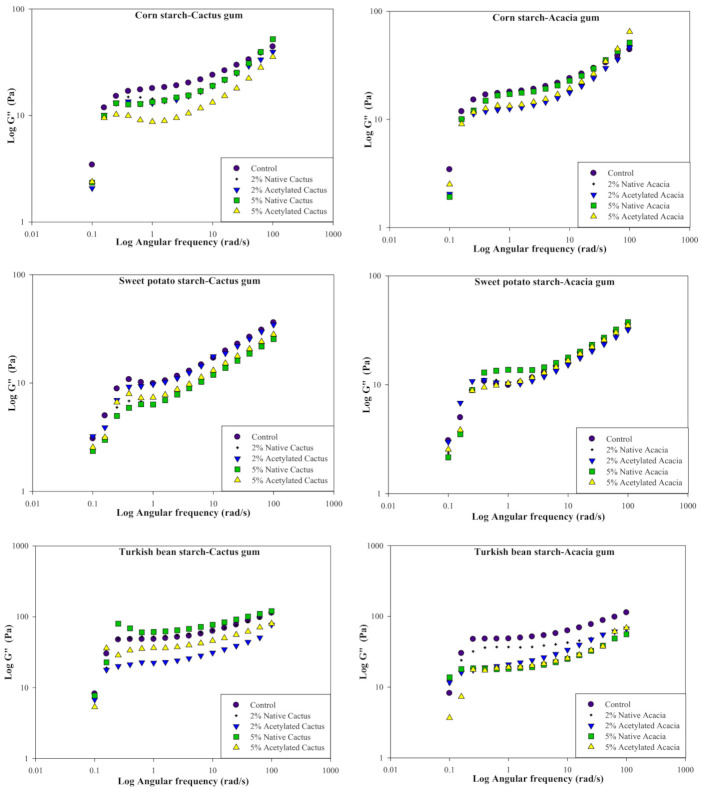
Effect of cactus and acacia gums on the storage modulus (Gʺ) of corn, sweet potato, and Turkish bean starches.

Figure 5 shows the loss or viscous modulus (G″) of all the starch dispersions. All of the starch dispersions had a lower G″ than G′, indicating that they were less viscous than the elastic ones. TBS dispersion with CG or AG, on the other hand, offered the highest G″ when compared to CS and SPS. For all samples with native or acetylated CG, a sharp rise in the G″ was seen at minor deformation in the case of CS. The G″ was greatest in the control sample without CS than in the presence of native or acetylated CG. The maize starch, on the other hand, showed a breakdown (Figure 5) with increasing deformation. However, for all dispersions with or without CG, a rise in the G″ was seen in conjunction with a rise in the angular frequency (ω). The viscous aspect of the dispersion was reduced with the addition of CG, with the dispersion with 5% acetylated CG being the closest to the horizontal axis, indicating poor starch-CG contact and greater interaction between CG molecules. This suggests that adding CG to the dispersions did not improve their viscous behavior. Similarly, with AG, the CS dispersions had a lower G″ than the control, with the sample with 5% acetylated AG having the lowest G″. However, in the presence of AG, there was a minor gap in the CS dispersion rheological profiles, indicating that the change in AG concentration did not cause large changes in their viscous behavior.

The existence of CG resulted in a very strong dependency on frequency for the SPS at first. The 5% native CG with SPS showed the most reliance, whereas the dispersions with acetylated CG showed the least. Large deformation imparted a rise in G″ for all dispersions, though not exponentially. Overall, there was a strong slope for the SPS with the CG. In the case of AG with SPS, the 5% acetylated dispersion had a larger G″, whilst the 2 percent AG (native or modified) had a lesser dependency on the applied deformation. The SPS dispersion with 2% acetylated AG, on the other hand, was unaffected by the deformation changes. Surprisingly, at the greatest angular frequency of 100 rad/s, all of the profiles overlapped. The total physical distance across profiles was less, indicating that the SPS and the AG interacted at a similar level.

TBS dispersions with CG, like other starches, offered the greatest change in G″ at tiny deformations, while increasing the angular frequency resulted in an increase in G″, with native 5% CG providing the highest value. The sample with 5 percent acetylated CG had the smallest change in the G″ of all the samples. TBS samples with native AG, on the other hand, performed better than TBS samples with acetylated AG. All of the TBS dispersions with AG, on the other hand, had a lower G″ than the control TBS dispersion. G″ grew as the angular frequency was raised, although TBS with CG functioned well and had a larger G″ than AG equivalents. Rivera-Corona et al., [36] found that adding Mexican CG to sweet sorghum starch dispersion resulted in lower G″ than G′.

### 3.7. Steady Shear Behaviors of Gels Prepared from Starch Gum Mixtures

**Figure 6:**
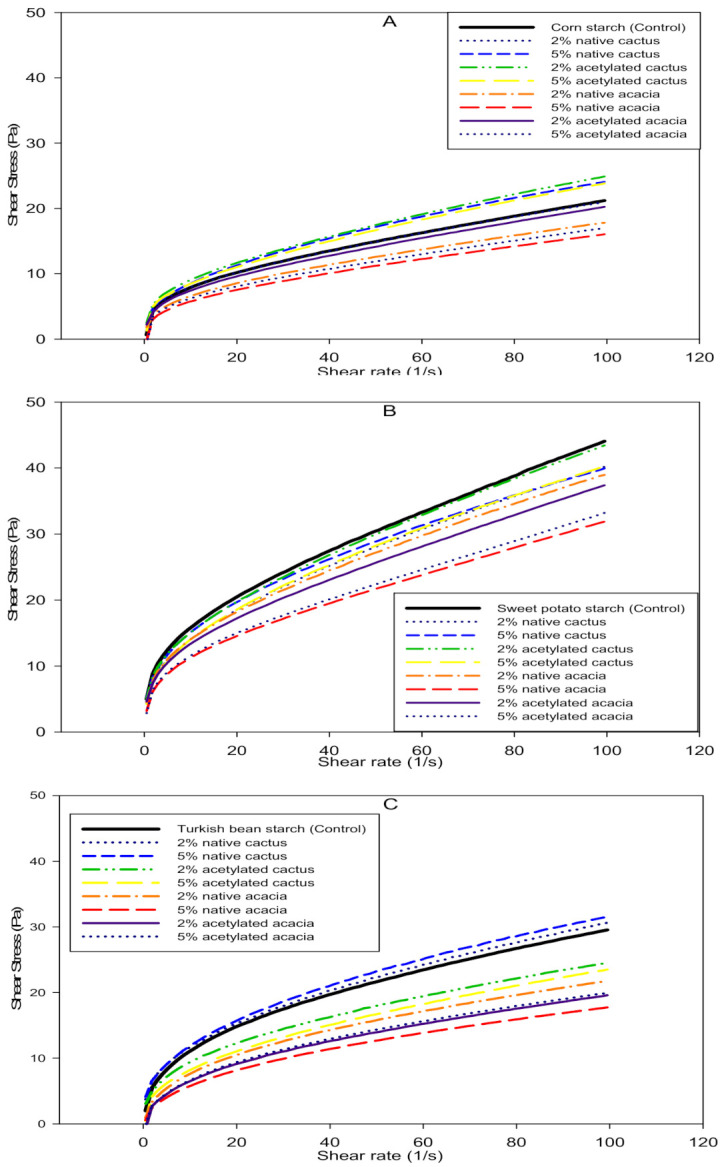
Shear rate (S^−1^) vs. shear stress (Pa) plots of corn (**A**), sweet potato (**B**), and Turkish bean (**C**) starches blends with cactus and acacia gums 50 °C.

A variety of additives are used in foods to improve their organoleptic properties. As a bulking agent, starch with various gums and mucilages is commonly used to provide body and texture to food gels. The shear behavior of gels varies depending on the additives and their level of interaction. In this study, three starches were combined with native and acetylated CG and AG: corn starch (CS), sweet potato starch (SPS), and Turkish bean starch (TBS), and their shear viscosity profiles are shown in Figure 6.

The apparent viscosity of all starch gels decreased as shear was increased, with profiles indicating time-dependent thixotropic behavior. All of the starch gels, with or without gums, showed a non-Newtonian shear thinning trend and yield stress in the shear stress vs. shear rate graphs. The thinning of starch gels under shear is caused by molecular chain alignment along the field of applied shear, which weakens the physical entanglement of polymeric chains at the starch-starch and starch-gum interfaces [48]. Among the starches, SPS had the lowest shear thinning compared to TBS and CS, with CS gels having the highest shear thinning. In the profiles, the yield stress depicts the interactive or overlapped structures in starch gels. This parameter serves as an indirect measure of the firmness of starch gels in the presence of other additives [49]. Without the addition of any gum, the gels with the highest yield stress were observed for the SPS, indicating firmer gels. In contrast, the SPS containing 5% acetylated AG had the lowest yield stress. However, SPS with CG produced firm gels with higher yield stress, whereas the presence of higher concentrations (5%) of AG produced softer gels.

The slant or slope of CS with 2% acetylated AG, on the other hand, was the highest, while gels with 5% native AG remained close to the horizontal axis. The highest shear thinning was observed for AG at higher concentrations (5%) and the lowest for 2% acetylated CG. At the start of shearing, the physical gap between starch profiles was close, but at 100 (s^−1^) shear rate, the gap was greater between starch gels with CG (native or modified) and also for the AG. However, among starches, CG remained close to control, whereas AG showed greater shear thinning. The starch gels with CG provided improved firmness, demonstrating a synergistic interaction between the starch and the CG. Higher shear thinning was observed with AG, whereas CG performed better than the control, improved gel rheology, and provided greater strength.

In the case of TBS, the highest slope was observed for gels containing 5% CG (native and acetylated), which also had higher yield stress and firmer gels than the control. Higher shear thinning was observed in the starch gels with AG, especially when a higher concentration was added. This implies that the AG interaction with TBS was comparable to that of the CS gels. The apparent gap between the profiles of control and TBS with AG, on the other hand, was larger, which could indicate partial phase separation of gums and starch. With a given change in shear rate, the overall change in shear stress was greater for the TBS. As a result, starch gels containing CG performed better under applied shear and may be capable of providing a cohesive structure for foods.

Similarly, all of the gels for CS with CG had a sharper slope and higher yield stress than the control. However, higher concentrations of AG in CS gels resulted in greater shear thinning than CG. Among the gums, AG retained the firmness of the gels better due to higher slope and lower shear thinning. CS gels containing 2% AG or CG, on the other hand, outperformed CS gels containing 5% AG or CG in terms of firmness. While native AG or CG interacted better with the starch chains in the CS gels and retained firmness better. As a result, it was possible to conclude that native and acetylated gums altered the functionality of the starch gels differently and exhibited different yield stress. Among all starches, SPS gels with native CG could provide firmer gels.

The flow behavior index (*n*) and consistency index (*k*) of starch gels were calculated using the power law model. The flow behavior index (*n*) indicates whether material shear thickening or thinning occurs under applied shear. While the consistency index (*k*) is an indirect measure of gel viscosity. All of the starch-gum blends exhibited non-Newtonian behavior because the *n* was less than one, and shear thinning was observed (Table 5). The non-Newtonian behavior of starch gels with gums depicts polymeric chain alignment in the field of applied force and physically weak interactions at starch-starch or starch-gum interfaces [48].

TBS gels had the highest shear thinning and lowest pseudoplasticity (lowest *n*), with *n* = 0.39. Temperatures ranging from 30 to 50 °C caused only minor changes in the pseudoplasticity of CS gels containing either native or acetylated AG and CG. However, for SPS gels, temperature changes reduced pseudoplasticity at all levels of native or modified AG and CG. The presence of CG and AG in the TBS gels, on the other hand, increased the n, indicating that shear had the least influence on the gel structure. The overall variation in the *n* was as follows: SPS > CS > TBS. This variation could be attributed to the SPS’s higher amylose content and intact granular structure [49].

The consistency index (*k*) measures the viscosity of starch gels with various gums. The increase in temperature resulted in a decrease in the *k* of all starch gels containing AG or CG. The SPS had the highest *k* value among the starches, with a value of 6.85 at 30 °C without the addition of gums (Table 5). On the other hand, the control CS gel had the lowest *k* (3.77), which decreased to 2.68 as the temperature increased from 30 to 50 °C. The addition of either native or acetylated CG increased the *k* values of CS, TBS, and SPS. With the addition of AG to the starch gels, the reduction in *k* was dramatic, especially at high levels of AG (5%).

When acetylated AG was present in the starch blends, the reduction in *k* was more pronounced than when native counterparts were present. Because of the lower total hydroxyl groups and the presence of acetyl groups on the sugar backbone, the acetylated AG had a weaker interaction with starches, resulting in a less viscous nature of starch blends. The CG remained better at controlling the *k* of the starch gels than the AG, as the overall *k* values for the starch/AG gels were lower than the starch/CG gels. An increase in gel temperature (30 to 50 °C) resulted in lower k due to greater mobility of the polymeric chains in the direction of applied shear. The extent of *k* reduction in gels of different starches could be attributed to their amylose contents and different levels of interaction with added gums.

### 3.8. Activation Energy Parameters of Gels Prepared from Starch-Gum Mixtures

The Arrhenius model was used to compute frequency factor (*µ_o_*) and activation energy (*Ea*), and the results are shown in Table 6. The correction coefficient (R^2^) remains sufficiently higher (0.75–0.99) for all estimations, confirming the model’s suitability for starch gels.

The frequency factor (*µ_o_*) is a constant that determines the abundance of energy in colliding molecules, which is required for a reaction to occur. Starch gels containing native AG and CG had a higher frequency factor (*µ_o_*) than starch gels containing modified gums. Mahmood et al. (2019) reported an increase in (*µ_o_*) of wheat flour in the presence of Cordia gum, either native or acetylated. The SPS gels with CG had the highest (*µ_o_*), followed by CS and TBS. The addition of AG to SPS gels resulted in a higher o than the gels containing CG. However, increasing shear (ramping up shears) reduced o for the same gels when compared to ramping down shear (from 100 to 0 s^−1^).

*Ea* represents the energy barrier that must be overcome in order to initiate the flow process in the gels [50]. The addition of acetylated CG gum to CS resulted in higher *Ea* than the native counterparts and the control. The gels with native cactus gum (2%) had the lowest *Ea*, indicating the least energy barrier, and lower temperatures can initiate the flow process. In the case of SPS, adding CG reduced the *Ea* for the gels. However, the control gel for SPS had the highest *Ea*. The acetylated CG blend with TBS, like the CS, had a higher *Ea* and temperature dependency, while native gums had the lowest (at 5%). As a result, the native and acetylated gums changed the level of interaction at the intra and intermolecular levels, resulting in different *Ea* for different starches. Lower *Ea* values indicate lower temperature dependence when compared to higher values. With the addition of CG, either native or acetylated, most of the starch gels displayed a similar order of *Ea* when the shear was ramped up or down. Overall, the starch gels with CG provided higher *Ea* as shear decreased from 100 to 0 s^−1^.

When compared to starch/CG mixtures, starch mixtures with AG, whether native or acetylated, increased *Ea*. The gels with TBS had the highest *Ea*, while the gels with SWS and native AG had the lowest at 2%. For the starch gels, increasing the level of AG resulted in a consistent pattern of increasing *Ea*. This implies that the presence of a higher gum concentration (5%) in the starch gels makes the viscosity less responsive to temperature change than a lower or no gum addition. More specifically, starch gels with a higher gum concentration (5%) provided greater *Ea* than their native counterparts. The *Ea* of starch gels with AG was higher when ramping down than when ramping up; higher *Ea* values were observed for profiles when shear was reduced from higher to lower. In contrast to the current study, a higher level of gum Cordia resulted in a lower *Ea* of wheat flour gels. Overall, the temperature dependency of starch gels with CG was lower than that of gels with AG, as lower *Ea* was observed for blends with CG. Again, the acetylated CG outperformed the native counterparts in terms of *Ea*. Except for SPS, native gums with starches provided higher *Ea* than acetylated gums in the case of AG. In a previous study, adding native gum Cordia to wheat flour resulted in higher *Ea* of the gels than acetylated wheat flour [32].

## 4. Conclusions

Locally extracted cactus and acacia gums were used to modify the different properties of corn, sweet potato, and Turkish bean starches. In the presence of both natural and acetylated gums, significant increases in starch gelatinization temperatures, enthalpies, peak viscosities, final viscosities, and setback viscosities were found. All starch gels’ apparent viscosity decreased as shear was increased, showing time-dependent thixotropic behavior. All starch gels, with or without gums, displayed a non-Newtonian shear thinning tendency, and the value of *n* for all samples was less than one. The *k* of all starch gels containing gums decreased when the temperature was increased. G′ and G′′ magnitudes were also affected by gum concentration. Both with and without gums, the Turkish bean starch was more stable in the two freeze-thaw cycles. The presence of all types of gums had a significant effect on the hardness, cohesiveness, and chewiness of gels.

## Figures and Tables

**Table 1:** Pasting properties of corn, sweet potato, and Turkish bean starch gels containing different levels of cactus and acacia gums.

	PV (cP)	FV (cP)	SB (cP)	PT (°C)
**Corn starch**				
**cactus gum**				
Control	2872 ± 20 ^bc^	2697 ± 24 ^b^	1038 ± 23 ^a^	75.51 ± 0.31 ^b^
Native 2%	2910 ± 07 ^b^	2544 ± 04 ^d^	874 ± 13 ^b^	76.67 ± 0.02 ^a^
Acetylated 2%	3013 ± 02 ^a^	2777 ± 03 ^a^	1058 ± 04 ^a^	76.82 ± 0.02 ^a^
Native 5%	2824 ± 11 ^d^	2405 ± 12 ^e^	775 ± 13 ^c^	77.08 ± 0.35 ^a^
Acetylated 5%	2848 ± 28 ^cd^	2594 ± 10 ^c^	1066 ± 45 ^a^	77.15 ± 0.37 ^a^
**acacia gum**				
Control	2872 ± 20 ^a^	2697 ± 24 ^a^	1038 ± 23 ^a^	75.51 ± 0.31 ^b^
Native 2%	2541 ± 23 ^c^	2505 ± 20 ^b^	932 ± 12 ^b^	76.03 ± 0.04 ^b^
Acetylated 2%	2690 ± 04 ^b^	2667 ± 11 ^a^	1061 ± 26 ^a^	75.53 ± 0.28 ^b^
Native 5%	1974 ± 14 ^e^	2162 ± 11 ^d^	755 ± 04 ^c^	76.75 ± 0.04 ^a^
Acetylated 5%	2145 ± 05 ^d^	2336 ± 04 ^c^	931 ± 12 ^b^	75.55 ± 0.26 ^b^
**Sweet potato starch**				
**cactus gum**				
Control	4047 ± 06 ^a^	2977 ± 22 ^c^	853 ± 38 ^b^	76.67 ± 0.02 ^c^
Native 2%	3882 ± 08 ^c^	3091 ± 26 ^a^	928 ± 27 ^a^	78.76 ± 0.28 ^b^
Acetylated 2%	3959 ± 25 ^b^	3079 ± 14 ^a^	891 ± 26 ^ab^	78.78 ± 0.31 ^b^
Native 5%	3813 ± 32 ^d^	3010 ± 25 ^b^	848 ± 13 ^b^	77.38 ± 0.72 ^c^
Acetylated 5%	3752 ± 15 ^e^	3011 ± 11 ^b^	894 ± 06 ^ab^	79.96 ± 0.01 ^a^
**acacia gum**				
Control	4047 ± 06 ^a^	2977 ± 22 ^a^	853 ± 38 ^b^	76.67 ± 0.02 ^c^
Native 2%	3154 ± 03 ^c^	2628 ± 04 ^d^	832 ± 06 ^b^	77.15 ± 0.34 ^b^
Acetylated 2%	3376 ± 20 ^b^	2886 ± 05 ^b^	970 ± 21 ^a^	76.73 ± 0.04 ^c^
Native 5%	2255 ± 04 ^d^	2154 ± 03 ^e^	780 ± 11 ^c^	76.74 ± 0.04 ^c^
Acetylated 5%	2625 ± 24 ^e^	2664 ± 07 ^c^	962 ± 18 ^a^	77.60 ± 0.04 ^a^
**Turkish bean starch**				
**cactus gum**				
Control	4430 ± 19 ^a^	5259 ± 45 ^a^	2592 ± 135 ^a^	71.77 ± 0.02 ^d^
Native 2%	4139 ± 33 ^b^	4797 ± 32 ^c^	2105 ± 26 ^b^	72.25 ± 0.35 ^cd^
Acetylated 2%	4163 ± 14 ^b^	4907 ± 25 ^b^	2117 ± 25 ^b^	72.63 ± 0.02 ^bc^
Native 5%	3709 ± 14 ^c^	4393 ± 23 ^e^	1948 ± 43 ^c^	73.03 ± 0.37 ^b^
Acetylated 5%	3726 ± 03 ^c^	4530 ± 13 ^d^	1916 ± 11 ^c^	74.27 ± 0.02 ^a^
**acacia gum**				
Control	4430 ± 19 ^a^	5259 ± 45 ^a^	2592 ± 135 ^a^	71.77 ± 0.02 ^bc^
Native 2%	3749 ± 22 ^c^	4622 ± 37 ^c^	2259 ± 45 ^b^	71.43 ± 0.41 ^c^
Acetylated 2%	3953 ± 65 ^b^	4959 ± 173 ^b^	2323 ± 110 ^b^	71.45 ± 0.31 ^c^
Native 5%	2682 ± 06 ^e^	3352 ± 34 ^e^	1497 ± 16 ^d^	72.67 ± 0.02 ^a^
Acetylated 5%	3186 ± 04 ^d^	4135 ± 14 ^d^	1899 ± 23 ^c^	72.28 ± 0.26 ^ab^

Means with same letters in columns for a particular gum (cactus or acacia) under different starches (corn, sweet potato, or Turkish bean) are non-significantly different from each other. Control = 100% starch; PV = Peak viscosity; cP = centipoise; FV = Final viscosity; SB = Setback viscosity; PT = Pasting temperature.

**Table 2:** Thermal properties of corn, sweet potato, and Turkish bean starch gels containing different levels of cactus and acacia gums.

	∆H (J/g)	*To* (°C)	*Tp* (°C)
**Corn starch**			
**cactus gum**			
Control	12.36 ± 0.41 ^b^	66.18 ± 0.02 ^d^	71.14 ± 0.10 ^e^
Native 2%	13.31 ± 0.17 ^a^	68.75 ± 0.07 ^c^	73.49 ± 0.08 ^d^
Acetylated 2%	12.19 ± 0.11 ^b^	69.00 ± 0.02 ^c^	73.82 ± 0.05 ^c^
Native 5%	12.65 ± 0.04 ^b^	69.59 ± 0.46 ^b^	74.58 ± 0.24 ^b^
Acetylated 5%	12.16 ± 0.23 ^b^	70.52 ± 0.09 ^a^	75.30 ± 0.15 ^a^
**acacia gum**			
Control	12.36 ± 0.41 ^a^	66.18 ± 0.02 ^d^	71.14 ± 0.10 ^d^
Native 2%	12.66 ± 0.14 ^a^	66.28 ± 0.01 ^c^	71.25 ± 0.16 ^d^
Acetylated 2%	12.57 ± 0.08 ^a^	66.96 ± 0.07 ^b^	71.78 ± 0.01 ^b^
Native 5%	10.10 ± 0.11 ^c^	66.31 ± 0.01 ^c^	71.54 ± 0.04 ^c^
Acetylated 5%	11.03 ± 0.25 ^b^	67.28 ± 0.03 ^a^	72.30 ± 0.09 ^a^
**Sweet potato starch**			
**cactus gum**			
Control	12.87 ± 0.30 ^c^	67.91 ± 0.13 ^d^	79.53 ± 0.16 ^c^
Native 2%	14.03 ± 0.08 ^b^	69.57 ± 0.25 ^c^	80.72 ± 0.22 ^b^
Acetylated 2%	12.86 ± 0.14 ^c^	70.48 ± 0.13 ^b^	80.43 ± 0.14 ^b^
Native 5%	15.18 ± 0.03 ^a^	70.70 ± 0.01 ^b^	82.24 ± 0.01 ^a^
Acetylated 5%	12.65 ± 0.12 ^c^	71.58 ± 0.08 ^a^	82.61 ± 0.26 ^a^
**acacia gum**			
Control	12.87 ± 0.30 ^a^	67.91 ± 0.13 ^d^	79.53 ± 0.16 ^a^
Native 2%	12.23 ± 0.14 ^b^	68.93 ± 0.24 ^b^	79.43 ± 0.03 ^a^
Acetylated 2%	12.13 ± 0.03 ^b^	69.25 ± 0.04 ^a^	79.74 ± 0.06 ^a^
Native 5%	11.80 ± 0.17 ^b^	68.61 ± 0.09 ^c^	79.87 ± 0.01 ^a^
Acetylated 5%	10.91 ± 0.20 ^c^	68.75 ± 0.02 ^bc^	79.57 ± 0.47 ^a^
**Turkish bean starch**			
**cactus gum**			
Control	11.25 ± 0.12 ^a^	59.62 ± 0.07 ^c^	69.65 ± 0.02 ^e^
Native 2%	9.00 ± 0.38 ^c^	60.86 ± 0.50 ^b^	70.93 ± 0.09 ^d^
Acetylated 2%	10.67 ± 0.04 ^b^	60.91 ± 0.16 ^b^	71.52 ± 0.28 ^c^
Native 5%	10.29 ± 0.05 ^b^	62.47 ± 0.02 ^a^	72.06 ± 0.03 ^b^
Acetylated 5%	9.10 ± 0.17 ^c^	61.55 ± 0.92 ^ab^	72.62 ± 0.13 ^a^
**acacia gum**			
Control	11.25 ± 0.12 ^b^	59.62 ± 0.07 ^c^	69.65 ± 0.02 ^b^
Native 2%	9.84 ± 0.09 ^c^	60.27 ± 0.03 ^b^	69.65 ± 0.04 ^b^
Acetylated 2%	9.69 ± 0.23 ^c^	59.66 ± 0.17 ^c^	69.64 ± 0.04 ^b^
Native 5%	10.98 ± 0.22 ^b^	60.49 ± 0.02 ^a^	69.25 ± 0.01 ^c^
Acetylated 5%	11.89 ± 0.03 ^a^	60.50 ± 0.02 ^a^	70.06 ± 0.04 ^a^

Means with same letters in columns for a particular gum (cactus or acacia) under different starches (corn, sweet potato, or Turkish bean) are non-significantly different from each other. Control = 100% starch; ∆H = Heat capacity; J/g = Jouls/gram; *To* = Onset temperature; *Tp* = Peak temperature.

**Table 3:** Percent syneresis from corn, sweet potato, and Turkish bean starch gels containing different levels of cactus and acacia gums.

	Day 4 (%)	Day 8 (%)	Total (%)
**Corn starch**			
**cactus gum**			
Control	13.41 ± 0.13 ^c^	3.38 ± 0.29 ^a^	16.79 ± 0.20 ^b^
Native 2%	12.66 ± 0.47 ^c^	3.33 ± 0.19 ^a^	15.99 ± 0.64 ^bc^
Acetylated 2%	11.48 ± 0.83 ^d^	0.12 ± 0.02 ^d^	11.59 ± 0.84 ^d^
Native 5%	17.12 ± 0.19 ^a^	1.51 ± 0.16 ^b^	18.63 ± 0.36 ^a^
Acetylated 5%	15.19 ± 0.61 ^b^	0.25 ± 0.04 ^c^	15.44 ± 0.63 ^c^
**acacia gum**			
Control	13.41 ± 0.13 ^c^	3.38 ± 0.29 ^a^	16.79 ± 0.20 ^b^
Native 2%	10.91 ± 1.01 ^d^	0.00 ± 0.00 ^b^	10.91 ± 1.01 ^c^
Acetylated 2%	6.30 ± 0.91 ^e^	0.00 ± 0.00 ^b^	6.30 ± 0.91 ^d^
Native 5%	16.46 ± 1.82 ^b^	0.00 ± 0.00 ^b^	16.46 ± 1.82 ^b^
Acetylated 5%	20.17 ± 0.62 ^a^	0.00 ± 0.00 ^b^	20.17 ± 0.62 ^a^
**Sweet potato starch**			
**cactus gum**			
Control	3.71 ± 0.45 ^a^	0.00 ± 0.00 ^d^	3.71 ± 0.45 ^a^
Native 2%	1.51 ± 0.04 ^b^	0.00 ± 0.00 ^d^	1.51 ± 0.04 ^c^
Acetylated 2%	1.71 ± 0.23 ^b^	0.12 ± 0.01 ^c^	1.84 ± 0.23 ^c^
Native 5%	3.14 ± 0.33 ^a^	0.15 ± 0.04 ^b^	3.30 ± 0.31 ^b^
Acetylated 5%	0.00 ± 0.00 ^c^	0.23 ± 0.03 ^a^	0.23 ± 0.03 ^d^
**acacia gum**			
Control	3.71 ± 0.45 ^b^	0.00 ± 0.00 ^c^	3.71 ± 0.45 ^c^
Native 2%	0.00 ± 0.00 ^d^	1.63 ± 0.09 ^b^	1.63 ± 0.09 ^d^
Acetylated 2%	3.44 ± 0.44 ^b^	0.00 ± 0.00 ^c^	3.44 ± 0.44 ^c^
Native 5%	2.48 ± 0.32 ^c^	4.78 ± 0.14 ^a^	7.25 ± 0.22 ^a^
Acetylated 5%	5.55 ± 0.41 ^a^	0.00 ± 0.00 ^c^	5.55 ± 0.41 ^b^
**Turkish bean starch**			
**cactus gum**			
Control	0.14 ± 0.05 ^c^	0.00 ± 0.00 ^d^	0.14 ± 0.05 ^e^
Native 2%	0.00 ± 0.00 ^d^	0.70 ± 0.07 ^c^	0.70 ± 0.07 ^d^
Acetylated 2%	2.82 ± 0.19 ^a^	1.35 ± 0.15 ^b^	4.17 ± 0.26 ^a^
Native 5%	0.00 ± 0.00 ^d^	1.52 ± 0.05 ^b^	1.52 ± 0.05 ^c^
Acetylated 5%	0.54 ± 0.09 ^b^	2.26 ± 0.19 ^a^	2.79 ± 0.24 ^b^
**acacia gum**			
Control	0.14 ± 0.05 ^e^	0.00 ± 0.00 ^e^	0.14 ± 0.05 ^e^
Native 2%	1.48 ± 0.07 ^d^	2.29 ± 0.14 ^b^	3.77 ± 0.21 ^d^
Acetylated 2%	2.90 ± 0.19 ^c^	1.37 ± 0.04 ^d^	4.27 ± 0.20 ^c^
Native 5%	4.93 ± 0.13 ^a^	2.80 ± 0.31 ^a^	7.37 ± 0.44 ^a^
Acetylated 5%	3.90 ± 0.59 ^b^	1.65 ± 0.15 ^c^	5.55 ± 0.55 ^b^

Means with same letters in columns for a particular gum (cactus or acacia) under different starches (corn, sweet potato, or Turkish bean) are non-significantly different from each other.

**Table 4:** Textural properties of corn, sweet potato, and Turkish bean starch gels containing different levels of cactus and acacia gums.

	Hardness (g)	Cohesiveness	Gumminess (g)
**Corn starch**			
**cactus gum**			
Control	214 ± 4.50 ^a^	0.47 ± 0.00 ^b^	100 ± 1.23 ^a^
Native 2%	177 ± 3.30 ^b^	0.47 ± 0.02 ^b^	83 ± 4.33 ^b^
Acetylated 2%	176 ± 3.40 ^b^	0.47 ± 0.01 ^b^	83 ± 2.09 ^b^
Native 5%	165 ± 4.55 ^c^	0.49 ± 0.02 ^a^	81 ± 6.24 ^b^
Acetylated 5%	147 ± 4.50 ^d^	0.46 ± 0.00 ^c^	67 ± 1.52 ^c^
**acacia gum**			
Control	214 ± 4.50 ^a^	0.47 ± 0.00 ^a^	100 ± 1.23 ^a^
Native 2%	176 ± 4.32 ^b^	0.44 ± 0.02 ^c^	77 ± 1.25 ^b^
Acetylated 2%	155 ± 2.87 ^d^	0.46 ± 0.02 ^b^	71 ± 2.06 ^c^
Native 5%	161 ± 2.49 ^c^	0.43 ± 0.02 ^d^	69 ± 3.11 ^d^
Acetylated 5%	127 ± 7.13 ^e^	0.46 ± 0.01 ^b^	59 ± 1.93 ^e^
**Sweet potato starch**			
**cactus gum**			
Control	154 ± 6.98 ^a^	0.71 ± 0.06 ^a^	109 ± 4.45 ^a^
Native 2%	108 ± 6.16 ^b^	0.67 ± 0.01 ^b^	73 ± 4.22 ^d^
Acetylated 2%	153 ± 7.36 ^a^	0.67 ± 0.04 ^b^	102 ± 1.23 ^b^
Native 5%	88 ± 10.42 ^c^	0.71 ± 0.01 ^a^	63 ± 7.84 ^e^
Acetylated 5%	153 ± 7.36 ^a^	0.64 ± 0.02 ^c^	89 ± 3.34 ^c^
**acacia gum**			
Control	154 ± 6.98 ^d^	0.71 ± 0.06 ^a^	109 ± 4.45 ^b^
Native 2%	176 ± 2.62 ^c^	0.51 ± 0.01 ^c^	90 ± 0.53 ^c^
Acetylated 2%	197 ± 11.67 ^b^	0.55 ± 0.02 ^b^	109 ± 5.36 ^b^
Native 5%	229 ± 6.27 ^a^	0.52 ± 0.01 ^c^	118 ± 5.97 ^a^
Acetylated 5%	175 ± 7.32 ^c^	0.52 ± 0.02 ^c^	91 ± 2.85 ^c^
**Turkish bean starch**			
**cactus gum**			
Control	852 ± 12.71 ^a^	0.33 ± 0.01 ^d^	284 ± 6.61 ^a^
Native 2%	691 ± 29.53 ^b^	0.42 ± 0.02 ^b^	287 ± 3.74 ^a^
Acetylated 2%	647 ± 27.05 ^d^	0.37 ± 0.01 ^c^	237 ± 3.26 ^c^
Native 5%	493 ± 31.46 ^e^	0.53 ± 0.02 ^a^	260 ± 24.23 ^b^
Acetylated 5%	567 ± 10.42 ^c^	0.41 ± 0.02 ^b^	230 ± 9.81 ^c^
**acacia gum**			
Control	852 ± 12.71 ^a^	0.33 ± 0.01 ^c^	284 ± 6.61 ^b^
Native 2%	663 ± 7.48 ^c^	0.38 ± 0.01 ^b^	250 ± 2.12 ^c^
Acetylated 2%	720 ± 32.15 ^b^	0.50 ± 0.02 ^a^	360 ± 27.39 ^a^
Native 5%	473 ± 9.18 ^d^	0.37 ± 0.00 ^b^	173 ± 1.42 ^e^
Acetylated 5%	472 ± 14.88 ^d^	0.38 ± 0.01 ^b^	179 ± 2.00 ^d^

Means with same letters in columns for a particular gum (cactus or acacia) under different starches (corn, sweet potato, or Turkish bean) are non-significantly different from each other. Control = 100% starch; g = grams.

**Table 5:** Steady shear parameters (Power law model) of corn, sweet potato, and Turkish bean starches containing cactus and acacia gums.

		*n*			*k* (Pa. s^n^)	
Temp	30 °C	40 °C	50 °C	30 °C	40 °C	50 °C
**Corn starch**						
**cactus gum**						
Control	0.46 ± 0.00	0.45 ± 0.00	0.44 ± 0.00	3.77 ± 0.00	3.33 ± 0.07	2.68 ± 0.03
Native 2%	0.46 ± 0.01	0.44 ± 0.01	0.44 ± 0.01	3.61 ± 0.02	3.17 ± 0.02	2.74 ± 0.03
Acetylated 2%	0.45 ± 0.01	0.45 ± 0.00	0.45 ± 0.01	4.50 ± 0.05	3.85 ± 0.03	2.97 ± 0.02
Native 5%	0.45 ± 0.01	0.45 ± 0.01	0.45 ± 0.00	4.00 ± 0.03	3.55 ± 0.02	2.84 ± 0.02
Acetylated 5%	0.46 ± 0.02	0.46 ± 0.01	0.46 ± 0.01	4.18 ± 0.33	3.61 ± 0.28	2.95 ± 0.17
**acacia gum**						
Control	0.46 ± 0.00	0.45 ± 0.00	0.44 ± 0.00	3.77 ± 0.00	3.33 ± 0.07	2.68 ± 0.03
Native 2%	0.46 ± 0.00	0.44 ± 0.01	0.44 ± 0.01	3.19 ± 0.16	2.86 ± 0.12	2.38 ± 0.06
Acetylated 2%	0.46 ± 0.01	0.44 ± 0.01	0.45 ± 0.01	3.74 ± 0.16	3.42 ± 0.15	2.56 ± 0.08
Native 5%	0.46 ± 0.00	0.45 ± 0.01	0.46 ± 0.01	2.85 ± 0.03	2.52 ± 0.02	1.91 ± 0.01
Acetylated 5%	0.46 ± 0.00	0.45 ± 0.01	0.45 ± 0.01	3.02 ± 0.06	2.68 ± 0.05	2.05 ± 0.06
**Sweet potato starch**						
**cactus gum**						
Control	0.50 ± 0.01	0.47 ± 0.00	0.46 ± 0.01	6.85 ± 0.08	6.24 ± 0.02	5.12 ± 0.14
Native 2%	0.50 ± 0.01	0.47 ± 0.01	0.47 ± 0.01	5.89 ± 0.13	5.65 ± 0.01	4.71 ± 0.18
Acetylated 2%	0.49 ± 0.01	0.46 ± 0.00	0.47 ± 0.01	6.44 ± 0.03	6.14 ± 0.18	4.17 ± 0.01
Native 5%	0.47 ± 0.01	0.43 ± 0.01	0.43 ± 0.01	6.69 ± 0.01	6.64 ± 0.05	5.57 ± 0.12
Acetylated 5%	0.48 ± 0.01	0.44 ± 0.00	0.46 ± 0.01	6.12 ± 0.06	5.98 ± 0.07	4.95 ± 0.41
**acacia gum**						
Control	0.50 ± 0.01	0.47 ± 0.00	0.46 ± 0.01	6.85 ± 0.08	6.24 ± 0.02	5.12 ± 0.14
Native 2%	0.49 ± 0.01	0.45 ± 0.01	0.47 ± 0.02	5.65 ± 0.03	5.63 ± 0.03	4.47 ± 0.12
Acetylated 2%	0.50 ± 0.00	0.48 ± 0.01	0.47 ± 0.01	5.62 ± 0.24	5.16 ± 0.09	4.25 ± 0.04
Native 5%	0.49 ± 0.01	0.47 ± 0.01	0.46 ± 0.01	4.99 ± 0.29	4.77 ± 0.04	3.60 ± 0.07
Acetylated 5%	0.50 ± 0.01	0.49 ± 0.02	0.48 ± 0.01	5.09 ± 0.12	4.53 ± 0.15	3.56 ± 0.02
**Turkish bean starch**						
**cactus gum**						
Control	0.39 ± 0.01	0.40 ± 0.00	0.42 ± 0.01	6.45 ± 0.04	5.30 ± 0.01	4.23 ± 0.15
Native 2%	0.39 ± 0.00	0.40 ± 0.00	0.43 ± 0.01	6.40 ± 0.09	5.50 ± 0.02	4.19 ± 0.07
Acetylated 2%	0.39 ± 0.02	0.41 ± 0.02	0.43 ± 0.01	5.52 ± 0.29	4.58 ± 0.17	3.58 ± 0.06
Native 5%	0.40 ± 0.01	0.41 ± 0.02	0.43 ± 0.01	6.41 ± 0.20	5.57 ± 0.29	4.22 ± 0.15
Acetylated 5%	0.43 ± 0.01	0.43 ± 0.01	0.46 ± 0.01	5.02 ± 0.27	4.01 ± 0.12	2.84 ± 0.09
**acacia gum**						
Control	0.39 ± 0.01	0.40 ± 0.00	0.42 ± 0.01	6.45 ± 0.04	5.30 ± 0.01	4.23 ± 0.15
Native 2%	0.40 ± 0.01	0.42 ± 0.01	0.46 ± 0.01	4.82 ± 0.36	3.92 ± 0.31	2.82 ± 0.22
Acetylated 2%	0.41 ± 0.00	0.43 ± 0.01	0.49 ± 0.01	4.33 ± 0.28	3.41 ± 0.33	2.31 ± 0.16
Native 5%	0.40 ± 0.01	0.42 ± 0.01	0.40 ± 0.01	4.82 ± 0.36	3.98 ± 0.31	2.82 ± 0.22
Acetylated 5%	0.40 ± 0.01	0.43 ± 0.01	0.47 ± 0.01	4.05 ± 0.11	3.26 ± 0.08	2.24 ± 0.01

Control = 100% starch; *n* = flow behavior index (dimensionless); *k* = consistency coefficient (Pa.s^n^); Pa.s^n^ = Pascal seconds.

**Table 6:** Activation energy parameters of corn, sweet potato, and Turkish bean starches containing cactus and acacia gums.

Starch blends	Upward curves			Downward curves		
	*µ_°_* (Pa. s^n^)	*E_a_* (J/mol K^−1^)	R^2^	*µ_°_* (Pa. s^n^)	*E_a_* (J/mol K^−1^)	R^2^
**Corn starch**						
**cactus gum**						
Control	6.5660 × 10^−5^	13,928	0.90	5.2235 × 10^−3^	9613	0.99
Native 2%	6.7297 × 10^−4^	11,237	0.99	4.7466 × 10^−3^	9564	0.99
Acetylated 2%	6.7499 × 10^−6^	16,870	0.97	0.0209	8264	0.99
Native 5%	7.7757 × 10 ^−5^	13,897	0.96	2.3973 × 10^−3^	10,396	0.99
Acetylated 5%	6.2189 × 10^−5^	14,233	0.98	3.5838 × 10^−3^	10,086	0.99
**acacia gum**						
Native 2%	3.0076 × 10^−4^	11,829	0.97	4.2491 × 10^−3^	9423	0.99
Acetylated 2%	1.9258 × 10^−5^	15,291	0.94	4.2323 × 10^−3^	9867	0.99
Native 5%	4.3295 × 10^−6^	16,225	0.94	1.5690 × 10^−3^	10,262	0.99
Acetylated 5%	7.2538 × 10^−6^	15,805	0.94	2.3039 × 10^−3^	9979	0.99
**Sweet potato starch**						
**cactus gum**						
Control	1.7514 × 10^−3^	11,844	0.95	3.7718 × 10^−3^	25,471	0.99
Native 2%	0.0167	9005	0.87	6.7227 × 10^−7^	32,684	0.97
Acetylated 2%	1.1673 × 10^−3^	12,173	0.85	8.4015 × 10^−4^	26,114	0.99
Native 5%	0.0995	7386	0.78	1.4024 × 10^−3^	24,360	0.97
Acetylated 5%	0.0274	8572	0.81	9.194 × 10^−10^	39,884	0.94
**acacia gum**						
Native 2%	0.0109	9401	0.75	0.1166	19,105	0.82
Acetylated 2%	1.8394 × 10^−3^	11,291	0.94	3.0236 × 10^−5^	28,492	0.99
Native 5%	2.6898 × 10^−4^	13,148	0.84	6.0044 × 10^−3^	20,900	0.90
Acetylated 5%	8.1363 × 10^−5^	14,460	0.95	7.3436 × 10^−8^	34,418	0.99
**Turkish bean starch**						
**cactus gum**						
Control	1.1689 × 10^−5^	17,177	0.97	0.0102	9883	0.99
Native 2%	1.1700 × 10^−5^	17,160	0.96	0.0267	8837	0.99
Acetylated 2%	6.5760 × 10^−7^	19,939	0.97	1.4140 × 10^−3^	11,702	0.99
Native 5%	1.3628 × 10^−5^	17,006	0.96	7.5045 × 10^−3^	10,279	0.99
Acetylated 5%	2.7760 × 10^−8^	23,166	0.98	3.7411 × 10^−3^	10,265	0.97
**acacia gum**						
Native 2%	9.0287 × 10^−8^	21,785	0.96	1.4239 × 10^−3^	11,296	0.99
Acetylated 2%	2.3409 × 10^−9^	25,514	0.97	1.0008 × 10^−4^	13,983	0.99
Native 5%	2.3082 × 10^−10^	27,688	0.96	7.9285 × 10^−6^	16,432	0.99
Acetylated 5%	7.1332 × 10^−9^	24,131	0.97	2.7136 × 10^−4^	12,676	0.99

Control = 100% starch; *μ*_o_ (Pa s^n^) = is the frequency factor at a reference temperature (30, 40 and 50 °C); *Ea* = activation energy (J/mol K^−1^).

## Data Availability

Not applicable.

## Sample Availability

Samples of the compounds are not available from the authors.

## References

[1] Rosell C.M., Rojas J.A., De Barber C.B. (2001). Influence of hydrocolloids on dough rheology and bread quality. Food Hydrocoll..

[2] Hoefler A.C. (2004). Hydrocolloids.

[3] Funami T. (2011). Next target for food hydrocolloid studies: Texture design of foods using hydrocolloid technology. Food Hydrocoll..

[4] Guarda A., Rosell C.M., Benedito C., Galotto M.J. (2004). Different hydrocolloids as bread improvers and antistaling agents. Food Hydrocoll..

[5] Tran T., Thitipraphunkul K., Piyachomkwan K., Sriroth K. (2008). Effect of starch modifications and hydrocolloids on freezable water in cassava starch systems. Starch-Stärke.

[6] Asghar A., Anjum F.M., Butt M.S., Tariq M.W., Hussain S. (2007). Rheological and storage effect of hydrophillic gums on the quality of frozen dough pizza. Food Sci. Technol. Res..

[7] Christianson D., Hodge J., Osborne D., Detroy R.W. (1981). Gelatinization of wheat starch as modified by xanthan gum, guar gum, and cellulose gum. Cereal Chem..

[8] Rojas J.A., Rosell C.M., De Barber C.B. (1999). Pasting properties of different wheat flour-hydrocolloid systems. Food Hydrocoll..

[9] Toufeili I., Ismail B., Shadarevian S., Baalbaki R., Khatkar B., Bell A., Schofield J. (1999). The role of gluten proteins in the baking of arabic bread. J. Cereal Sci..

[10] Hebeda R., Zobel H. (1996). Baked Goods Freshness: Technology, Evaluation, and Inhibition of Staling.

[11] Hussain S. (2015). Native rice starch and linseed gum blends: Effect on the pasting, thermal and rheological properties. Czech J. Food Sci..

[12] Phillips G.O., Williams P.A. (2009). Handbook of Hydrocolloids.

[13] Gomez-Diaz D., Navaza J.M. (2003). Comments about rheological effects of food hydrocolloids addition. J. Food Agric. Environ..

[14] Rosell C.M., Yokoyama W., Shoemaker C. (2011). Rheology of different hydrocolloids-rice starch blends. Effect of successive heating-cooling cycles. Carbohydr. Polym..

[15] Achayuthakan P., Suphantharika M. (2008). Pasting and rheological properties of waxy corn starch as affected by guar gum and xanthan gum. Carbohydr. Polym..

[16] Alamri M.S., Mohamed A.A., Hussain S. (2012). Effect of okra gum on the pasting, thermal, and viscous properties of rice and sorghum starches. Carbohydr. Polym..

[17] Alamri M.S., Mohamed A.A., Hussain S. (2013). Effects of alkaline-soluble okra gum on rheological and thermal properties of systems with wheat or corn starch. Food Hydrocoll..

[18] Ali B.H., Ziada A., Blunden G. (2009). Biological effects of gum arabic: A review of some recent research. Food Chem. Toxicol..

[19] Baveja S. (1988). Examination of natural gums and mucilages as sustaining materials in tablets dosage forms. Indian J. Pharm. Sci..

[20] Fox D.I., Pichler T., Yeh D.H., Alcantar N.A. (2012). Removing heavy metals in water: The interaction of cactus mucilage and arsenate (As (V)). Environ. Sci. Technol..

[21] Pichler T., Young K., Alcantar N. (2012). Eliminating turbidity in drinking water using the mucilage of a common cactus. Water Sci. Technol.: Water Supply.

[22] Cardenas A., Higuera-Ciapara I., Goycoolea F. (1997). Rheology and aggregation of cactus (opuntia ficus-indica) mucilage in solution. J. Prof. Assoc. Cactus Dev..

[23] Synytsya A., Čopíková J., Matějka P., Machovič V. (2003). Fourier transform Raman and infrared spectroscopy of pectins. Carbohydr. Polym..

[24] Alba K., Laws A.P., Kontogiorgos V. (2015). Isolation and characterization of acetylated LM-pectins extracted from okra pods. Food Hydrocoll..

[25] Andrade L.A., Nunes C.A., Pereira J. (2015). Relationship between the chemical components of taro rhizome mucilage and its emulsifying property. Food Chem..

[26] Osman M.E., Menzies A.R., Martin B.A., Williams P.A., Phillips G.O., Baldwin T.C. (1995). Characterization of gum arabic fractions obtained by anion-exchange chromatography. Phytochemistry.

[27] Chatjigakis A., Pappas C., Proxenia N., Kalantzi O., Rodis P., Polissiou M. (1998). FT-IR spectroscopic determination of the degree of esterification of cell wall pectins from stored peaches and correlation to textural changes. Carbohydr. Polym..

[28] Njintang N.Y., Boudjeko T., Tatsadjieu L.N., Nguema-Ona E., Scher J., Mbofung C.M. (2014). Compositional, spectroscopic and rheological analyses of mucilage isolated from taro (*Colocasia esculenta* L. Schott) corms. J. Food Sci. Technol..

[29] Barka N., Abdennouri M., El Makhfouk M., Qourzal S. (2013). Biosorption characteristics of cadmium and lead onto eco-friendly dried cactus (Opuntia ficus indica) cladodes. J. Environ. Chem. Eng..

[30] Singh A., Geveke D.J., Yadav M.P. (2017). Improvement of rheological, thermal and functional properties of tapioca starch by using gum arabic. LWT.

[31] Chaisawang M., Suphantharika M. (2006). Pasting and rheological properties of native and anionic tapioca starches as modified by guar gum and xanthan gum. Food Hydrocoll..

[32] Mahmood K., Alamri M.S., Abdellatif M.A., Hussain S., Qasem A.A.A. (2018). Wheat flour and gum cordia composite system: Pasting, rheology and texture studies. Food Sci. Technol..

[33] Byars J.A., Singh M., Kenar J.A. (2017). Effect of hydrocolloids on functional properties of navy bean starch. Starch-Stärke.

[34] von Borries-Medrano E., Jaime-Fonseca M.R., Aguilar-Méndez M.A. (2019). Tapioca starch-galactomannan systems: Comparative studies of rheological and textural properties. Int. J. Biol. Macromol..

[35] Hussain S., Mohamed A.A., Alamri M.S., Ibraheem M.A., Qasem A.A.A., Shahzad S.A., Ababtain I.A. (2020). Use of gum cordia (cordia myxa) as a natural starch modifier; effect on pasting, thermal, textural, and rheological properties of corn starch. Foods.

[36] Rivera-Corona J.L., Rodríguez-González F., Rendón-Villalobos R., García-Hernández E., Solorza-Feria J. (2014). Thermal, structural and rheological properties of sorghum starch with cactus mucilage addition. LWT-Food Sci. Technol..

[37] Alamri M.S., Mohamed A., Hussain S., Xu J. (2012). Effect of Okra extract on properties of wheat, corn and rice starches. J. Food Agric. Environ..

[38] Pongsawatmanit R., Chantaro P., Nishinari K. (2013). Thermal and rheological properties of tapioca starch gels with and without xanthan gum under cold storage. J. Food Eng..

[39] Arocas A., Sanz T., Fiszman S. (2009). Improving effect of xanthan and locust bean gums on the freeze-thaw stability of white sauces made with different native starches. Food Hydrocoll..

[40] Pongsawatmanit R., Temsiripong T., Ikeda S., Nishinari K. (2006). Influence of tamarind seed xyloglucan on rheological properties and thermal stability of tapioca starch. J. Food Eng..

[41] da Silva Costa R.A., Bonomo R.C.F., Rodrigues L.B., Santos L.S., Veloso C.M. (2020). Improvement of texture properties and syneresis of arrowroot (Maranta arundinacea) starch gels by using hydrocolloids (guar gum and xanthan gum). J. Sci. Food Agric..

[42] Cheng H. (1980). Xanthan Gum and Locust Bean Gum in Confectionery Use. U.S. Patent.

[43] Kaur L., Singh J., Singh H., McCarthy O. (2009). Cassia gum: A novel galactomannan for use in starch based foods. Getreidetechnologie.

[44] Gheribi R., Khwaldia K. (2019). Cactus mucilage for food packaging applications. Coatings.

[45] Sati F., Qubbaj T. (2021). Impact of gum Arabic and cactus mucilage as potential coating substances combined with calcium chloride treatment on tomato (*Solanum lycopersicum* L.) fruit quality attributes under ambient storage conditions. Can. J. Plant Sci..

[46] Kim C., Lee S.P., Yoo B. (2006). Dynamic rheology of rice starch-galactomannan mixtures in the aging process. Starch-Stärke.

[47] Sudhakar V., Singhal R., Kulkarni P. (1996). Starch-galactomannan interactions: Functionality and rheological aspects. Food Chem..

[48] Yu H.-Y., Wang L., McCarthy K.L. (2016). Characterization of yogurts made with milk solids nonfat by rheological behavior and nuclear magnetic resonance spectroscopy. J. Food Drug Anal..

[49] Hussain S., Mohamed A.A., Alamri M.S., Saleh A., Ibraheem M.A., Abdo Qasem A.A., Shamlan G., Abatain I.A. (2021). Rheological, textural, and sensory properties of non-fat yogurt containing cress (*Lepidium sativum*) seed gum and various starches. Food Sci. Technol..

[50] Abdo Qasem A.A., Alamri M., Mohamed A., Hussain S., Mahmood K., Ibraheem M. (2017). High soluble-fiber pudding: Formulation, processing, texture and sensory properties. J. Food Process. Preserv..

